# Eye movement related brain responses to emotional scenes during free viewing

**DOI:** 10.3389/fnsys.2013.00041

**Published:** 2013-08-20

**Authors:** Jaana Simola, Jari Torniainen, Mona Moisala, Markus Kivikangas, Christina M. Krause

**Affiliations:** Cognitive Science/Cognitive Brain Research Unit, Institute of Behavioural Sciences, University of HelsinkiHelsinki, Finland

**Keywords:** attention, emotion, EEG, eye movements, co-registration, fixation-related potentials, free viewing, LPP

## Abstract

Emotional stimuli are preferentially processed over neutral stimuli. Previous studies, however, disagree on whether emotional stimuli capture attention preattentively or whether the processing advantage is dependent on allocation of attention. The present study investigated attention and emotion processes by measuring brain responses related to eye movement events while 11 participants viewed images selected from the International Affective Picture System (IAPS). Brain responses to emotional stimuli were compared between serial and parallel presentation. An “emotional” set included one image with high positive or negative valence among neutral images. A “neutral” set comprised four neutral images. The participants were asked to indicate which picture—if any—was emotional and to rate that picture on valence and arousal. In the serial condition, the event-related potentials (ERPs) were time-locked to the stimulus onset. In the parallel condition, the ERPs were time-locked to the first eye entry on an image. The eye movement results showed facilitated processing of emotional, especially unpleasant information. The EEG results in both presentation conditions showed that the LPP (“late positive potential”) amplitudes at 400–500 ms were enlarged for the unpleasant and pleasant pictures as compared to neutral pictures. Moreover, the unpleasant scenes elicited stronger responses than pleasant scenes. The ERP results did not support parafoveal emotional processing, although the eye movement results suggested faster attention capture by emotional stimuli. Our findings, thus, suggested that emotional processing depends on overt attentional resources engaged in the processing of emotional content. The results also indicate that brain responses to emotional images can be analyzed time-locked to eye movement events, although the response amplitudes were larger during serial presentation.

## Introduction

Real world scene viewing is an active process during which viewers select regions of scenes that will be processed in detail by prioritizing highly salient and unexpected stimuli at the expense of other stimuli and ongoing neural activity. Converging research evidence supports a processing advantage for emotional stimuli, indicating that humans are able to detect emotional content rapidly among other salient stimuli in order to activate motivational resources for approach or avoidance (Crawford and Cacioppo, 2002; Vuilleumier, 2005; Olofsson et al., 2008). Although there is a vast amount of research showing that attention is efficiently drawn toward emotional stimuli, the current theories disagree on the role of attention in emotional processing and the actual time course of attention and emotion processes.

One line of research suggests that emotional stimuli automatically activate brain regions largely independent of attentional control. For example, visual search studies propose that emotional detectors work preattentively by directing attention automatically toward threat without conscious effortful processing. These studies have shown that potentially threatening stimuli are found efficiently among neutral distractors (Öhman et al., 2001; Blanchette, 2006; Fox et al., 2007). EEG studies recording steady-state visual evoked potentials (ssVEPs) have also suggested that non-attended emotional information modulates brain activity independent of the focus of spatial attention. This modulation occurs especially when the emotional content is presented in the left visual field (Keil et al., 2005). A decrease in the amplitudes of the ssVEPs and in target detection rates have also been observed when the primary attentional task (detecting coherent motion of dots) is superimposed over pictures of emotional scenes as compared to neutral scenes (Hindi Attar et al., 2010). Taken together, these studies support the view that affective processing can occur without allocation of attentional resources, and that emotional processing precedes semantic processing (i.e., *the affective primacy hypothesis*).

The assumption that emotional stimuli capture attention automatically has been challenged by studies suggesting that prior to affective analysis, the features of objects must be integrated and the objects must be categorized and identified (reviewed in Cave and Batty, 2006; Storbeck et al., 2006). These studies support the *cognitive primacy hypothesis*, which states that identifying an object is a necessary prerequisite for evaluating its significance. For example, brain responses to emotional facial expressions have been shown to depend on sufficient attention resources being available to process the faces (Pessoa et al., 2002). These results demonstrate that responses to foveally presented emotional expression disappear when attention is directed in detecting the orientation of peripherally presented bars. Moreover, Holmes et al. (2003) have shown an enhanced positivity in event-related potentials (ERPs) as a response to fearful relative to neutral faces only when attention is directed toward the face stimuli, while the emotional expression effect is completely eliminated in trials where faces were unattended. The data by Acunzo and Henderson (2011) also failed to demonstrate any automatic “pop-out” effect of emotional content by showing no differences in latencies of the first fixations to emotional and neutral objects within scenes. However, once the emotional items were fixated, they held attention longer than neutral objects. In sum, these studies argue against the preattentive view of emotional processing. What they posit instead is that the detection and processing of emotional information depends on the current locus of spatial attention.

In addition to the opposing views about the automatic processing of emotional content, other studies support a more flexible view of automaticity (see Moors and De Houwer, 2006). Eye movement studies have shown that encoding of emotional valence can take place even when affective processing is not relevant for the task (i.e., when participants are supposed to report the semantic category of the images) (Calvo and Nummenmaa, 2007). Moreover, emotional pictures are more likely to be fixated earlier than neutral pictures (e.g., Calvo and Lang, 2004; Nummenmaa et al., 2006), even when participants are instructed to fixate the neutral image (Nummenmaa et al., 2006). These results suggest that processing of affective information is facilitated over perceptual and semantic information. However, the facilitation of affective responses by emotionally congruent primes depended on pre-exposure to the primes (Calvo and Nummenmaa, 2007; Calvo and Avero, 2008), suggesting that the degree of awareness of the unattended stimulus valence affects affective priming. Furthermore, a gradual increase in affective priming occurred when the parafoveal primes were pre-exposed foveally as compared to when the primes were pre-exposed parafoveally (Calvo and Nummenmaa, 2007). Studies have also shown that when the primary task is more difficult, automatic orienting to emotional stimuli diminishes (Calvo and Nummenmaa, 2007; Becker and Detweiler-Bedell, 2009). Moreover, the exogenous drive of attention to emotional content disappears when emotional items are embedded in a scene, a condition in which the foveal and perceptual load is high (Acunzo and Henderson, 2011). These findings support a view that emotional processing can be fast, involuntary and performed in parallel with unrelated foveal tasks, but that emotional processing is sensitive to regulatory attentional influences (Vuilleumier, 2005).

Neurophysiology and neuroimaging results demonstrate that selective attention in perception is mediated by enhanced processing in sensory pathways (Vuilleumier, 2005). Studies recording ERPs have shown that in addition to the early sensory components (e.g., N1/P1 and N2/P2), picture emotionality is reflected as an “early posterior negativity” (EPN) difference between emotional and neutral stimuli, and as an enhanced “late positive potential” (LPP) component during processing of affective as compared to neutral stimuli (reviewed in Olofsson et al., 2008). The LPP is a sustained P300-like component that has an onset at around 250 ms post-stimulus and a posterior midline scalp distribution (Hajcak and Olvet, 2008). Similar to the P300, which is larger for attended than unattended stimuli, the enhanced LPP reflects greater attention to emotional stimuli (Cuthbert et al., 2000; Schupp et al., 2000, 2007). Prior research indicates that emotional information is highly salient and therefore also detected in the visual periphery. Parafoveally/peripherally presented emotional stimuli modulate both eye movement (Nummenmaa et al., 2009; Coy and Hutton, 2012) and ERP responses. For example, a modulation of the early and late ERPs by picture emotionality occurred also when pictures were presented in the peripheral vision (up to 8° eccentricity) and with short exposure times that prevent saccadic eye movements (De Cesarei et al., 2009).

In the present study, we investigated the time course and role of attention in emotional processing. In particular, we were interested in how attention is directed to emotional content during a free viewing task. Previous ERP studies have used paradigms that investigate the neural responses to emotional visual stimuli presented in isolation and intervened with unnaturally long inter-stimulus intervals. Neuronal activity under free viewing may, however, differ significantly with what is observed under restrictive stimulus conditions. Thus, it is not clear how well the neural responses obtained in constrained experimental conditions could explain the responses under natural oculomotor behavior, because natural visual processing is often motivated by specific goals, or the internal states of the viewer (Maldonado et al., 2009).

There is a rapidly growing interest in the use of co-registration of eye movements and EEG to study brain mechanisms during free viewing (see Baccino, 2011). In the analysis of co-registered data (i.e., the eye-fixation-related potential, EFRP analysis), the EEG signal is segmented based on eye movement events. Previous research using co-registration of eye movements and EEG has reported corresponding ERP data during unconstrained viewing conditions as compared to serial visual presentation (SVP) (Hutzler et al., 2007; Dimigen et al., 2011). Co-registration studies have also shown that parafoveal processing affects the ERP-responses at current fixation in reading (Dimigen et al., 2012) and reading-like tasks (Baccino and Manunta, 2005; Simola et al., 2009). Moreover, an earlier onset of the N400 was observed during natural reading than in SVP, possibly due to the parafoveal preview obtained in natural reading (Dimigen et al., 2011). In scene perception, information around the current fixation can be acquired from a wider region than during reading (see Rayner and Castelhano, 2008). This is especially evident in studies investigating attention to emotional stimuli (De Cesarei et al., 2009; Nummenmaa et al., 2009; Coy and Hutton, 2012). The high saliency of parafoveal information may constrain the use of the co-registration technique in emotional scene perception tasks. Therefore, the second aim of the present study was to validate the co-registration technique when participants were exposed to emotional scenes. Previous studies using co-registration of eye movements and EEG have mainly considered word recognition and reading processes (Baccino and Manunta, 2005; Simola et al., 2009; Dimigen et al., 2011, 2012). To our knowledge, no previous research has used the co-registration technique during free viewing of emotional scenes.

The use of co-registration technique involves several technical challenges including, for example, (i) the artifacts in EEG recordings caused by eye movements, (ii) accurate hardware synchronization between the eye movement and EEG data sets, (iii) temporal overlap between background EEG and fixation evoked ERPs as well as the temporal overlap of potentials elicited by successive fixations, and (iv) the phase differences of ERP responses due to systematic differences in eye movement variables. However, previous research suggests that most of these technical problems appear to be solvable (see Dimigen et al., 2011; Kliegl et al., 2012). We will discuss later how these problems were minimized in the present setup. Despite the technical challenges, the co-registration technique provides a valuable tool to understand the relation between oculomotor and brain electrical signals during cognitive processing. Using eye movements to segment the brain potentials helps to study brain activity under self-paced perceptual and cognitive behavior during free viewing tasks. This is relevant because even though eye movements can provide with indicators of cognitive processing under naturalistic viewing conditions, the eye movement data do not inform us about the time course of underlying processes that occur within subsequent fixations. Further, the combination of eye movement and EEG methods allows possibilities to investigate both spatial and temporal aspects of visual attention simultaneously.

Attention to emotional stimuli, in the present study, was investigated by recording eye movement related ERP-responses while participants performed visual search tasks to determine whether a group of scenes were neutral or whether there was an emotional scene among the neutral scenes. The stimulus material was presented in two conditions. That is, the participants saw sets consisting of four images either serially or in parallel. An “emotional” set included one image with highly pleasant or unpleasant content among neutral images. A “neutral” set comprised of four neutral images. A visual search paradigm was selected because it is a typical setup used in the studies of emotional processing (Öhman et al., 2001; Flykt, 2005; Blanchette, 2006; Fox et al., 2007). In contrast to many previous ERP-studies investigating parafoveal/peripheral processing of emotional content (e.g., Rigoulot et al., 2008), participants were allowed to move their eyes freely across the stimulus images. This kind of task condition permitted a natural foveal load across fixations (see Acunzo and Henderson, 2011).

Previous studies suggest that differences in tasks and measures may influence the effects of attention to emotional stimuli (e.g., Lipp et al., 2004; Blanchette, 2006). To ensure a fair comparison of the results from different data sets and to allow within-participant comparisons between the two presentation conditions, for each participant we collected different data (i.e., behavioral, eye-tracking and ERP-measures) during the same recording session. In the parallel condition, participants' eye movements and EEG were recorded simultaneously. Eye movement recordings allowed a comparison of results to previous eye movement studies of emotional processing (Calvo and Lang, 2004; Nummenmaa et al., 2006, 2009). Importantly, co-registration of eye movements and ERP responses permitted the analysis of brain responses time-locked to eye movement events. In order to validate the co-registration technique, the responses from parallel presentation were compared to the results from the serial condition and previous findings from the SVP studies (reviewed in Olofsson et al., 2008). The expectation was that if co-registration of eye movements and EEG is a valid technique to measure responses to emotional scenes, similar responses would occur in both presentation conditions. That is, we expected the LPP as a response to emotional processing in the serial presentation as well as in the parallel presentation when the ERPs were time-locked to the first entry of the target image.

Further, the emotional information is likely to be processed, at least to a certain degree, before the eyes have landed on the region of the emotional content. In order to examine the time course of emotional processing (i.e., the detection and parafoveal processing of emotional content), the ERP responses in the parallel condition were also time-locked to the stimulus onset. Since covert attention may be allocated to the emotional content when eyes are directed elsewhere on the stimulus (see Calvo and Lang, 2004), peripheral attention to emotional stimuli was expected to become visible in the ERP responses before the eyes move to the target image.

Facilitated attention has been reported in association with both pleasant and unpleasant stimuli (e.g., Nummenmaa et al., 2009; Coy and Hutton, 2012). These studies support “the emotionality hypothesis” by showing that attention is drawn to emotional information despite its emotional valence. On the basis of existing studies (e.g., Calvo and Lang, 2004; Nummenmaa et al., 2006, 2009), we expected that in the parallel presentation condition both pleasant and unpleasant pictures would be attended faster and for longer durations than neutral stimuli. In both presentation conditions, attention to emotional stimuli was also expected to elicit increased LPP responses for pleasant and unpleasant as compared to neutral pictures.

In addition to the “emotionality hypothesis,” several studies have reported that the valence of the stimulus determines how fast it is likely to capture attention. These studies have found that attention is automatically drawn to negative information more strongly than to positive information (Ito et al., 1998; Crawford and Cacioppo, 2002; Smith et al., 2003). This phenomenon is referred to as “the negativity effect” (or “the negativity hypothesis”). The evaluation of threat (or fear) may be the underlying component of this mechanism, and it may have developed during evolution as a survival mechanism (Öhman et al., 2001; Carretié et al., 2009). Both behavioral and ERP-studies have found support for the negativity effect. For example, the results from recognition and recall memory tests suggested that negative stimuli were better memorized than positive or neutral regions of the scenes (Humphrey et al., 2012). Also, a larger and more sustained LPP was elicited by unpleasant than pleasant stimuli (Ito et al., 1998; Smith et al., 2003; Hajcak and Olvet, 2008). Based on earlier studies, we also expected a negativity effect reflected in facilitated eye movement and ERP responses to unpleasant than to pleasant stimuli.

## Materials and methods

### Participants

Eleven volunteers [right-handed, 6 female, mean age: 21.3 ± 1.27 (SD)] with normal or corrected to normal vision participated in the experiment. All participants provided a written informed consent and were informed about the possible provocative content of the stimuli prior to the experiment. The participants reported no history of mental illness or neurological injury and were not on medication.

### Stimuli

The stimuli were 160 images selected from the International Affective Picture System (IAPS) (Lang et al., 2008). From the stimulus material, trials consisting of four images were generated. In a *pleasant* trial, one of the four images depicted people experiencing positive affect. In an *unpleasant* trial, one of the images was unpleasant, and presented people suffering serious threat or harm. In a *neutral* trial, four neutral images, showing daily non-emotional activities, were presented. The stimulus groups were selected such that there was no overlap in IAPS normative valence ratings between the categories. Mean valence ratings with 9-point scales were as follows, pleasant: 7.2 ± 2.4 (SD), unpleasant: 2.0 ± 1.5 (SD), neutral: 6.0 ± 1.5 (SD). Mean arousal ratings per stimulus groups were the following: pleasant: 6.7 ± 2.0 (SD), unpleasant: 6.5 ± 1.9 (SD), neutral: 3.9 ± 2.3 (SD). Appendix A lists the images used in this study.

Stimulus size was 560 × 420 pixels and the images subtended 15° horizontally and 11.4° vertically. In the serial condition, the images were presented at the center of the screen. In the parallel condition, the image size was identical, and the stimuli were presented symmetrically in the centers of the quadrants of the screen. The closest corner of the image to the screen center was 4.32°. Stimuli were presented on a 22-inch screen with the screen resolution of 1680 × 1050 pixels.

Previous research shows that low-level saliency guides our eye movements when inspecting a scene. We calculated the low-level image properties for the stimuli, in order to control for the possibility that the effects of emotional valence on eye movements and EEG responses would merely be a result of differences in the low-level visual features between neutral and emotional images (see e.g., Delplangue et al., 2007). The complexity of the images was assessed in terms of the size of each compressed JPEG-image in kilobytes (Donderi, 2006). The percentage of the area covered by faces was assessed for each image using ImageJ software, since human faces capture attention especially effectively (Calvo and Lang, 2005). Moreover, the percentage of images containing human faces was calculated per emotional conditions. The brightness and saturation levels per pixel were calculated for each image, and the skewness (i.e., the lack of symmetry of the intensity value distributions) and kurtosis (i.e., the pointiness of the distribution) were assessed for each color layer (red, green and blue). The mean scores and standard deviations for the low-level image characteristics are presented in Table 1.

**Table 1:** **Mean scores (and standard deviations) of the low-level image features for the emotional and neutral stimuli**.

	**Pleasant**	**Unpleasant**	**Neutral**
Complexity	304.80 (67.36)	272.05 (126.14)	291.19 (91.61)
Face area (%)	2.78 (6.31)	5.23 (5.58)	2.13 (6.20)
Occurrence of faces (%)	0.67 (0.48)	0.68 (0.48)	0.21 (0.41)
Brightness	0.54 (0.17)	0.47 (0.16)	0.47 (0.14)
Skewness (R)	0.11 (0.94)	0.18 (0.88)	0.25 (0.75)
Skewness (G)	0.31 (0.97)	0.60 (0.82)	0.47 (0.80)
Skewness (B)	0.36 (1.31)	0.86 (0.79)	0.82 (1.04)
Kurtosis (R)	3.01 (1.55)	2.64 (1.35)	2.52 (1.30)
Kurtosis (G)	3.30 (2.40)	2.96 (1.83)	3.00 (1.92)
Kurtosis (B)	4.18 (4.83)	3.28 (2.42)	4.00 (3.45)
Saturation	0.43 (0.16)	0.52 (0.17)	0.51 (0.19)

A One-Way analysis of variance (ANOVA) showed no differences in image complexity, brightness, skewness or kurtosis between the three image categories (*p* > 0.05). The ANOVA showed that the percentage of images containing faces differed between the emotional conditions [*F*_(2, 153)_= 21.25, *p* < 0.001]. Follow-up *t*-tests suggested differences between unpleasant and neutral [*t*_(112)_= 5.43, *p* < 0.001] and between pleasant and neutral conditions [*t*_(117)_ = 5.52, *p* < 0.001], while pleasant and unpleasant conditions did not differ in the occurrence of face images. However, it should be noted that the ANOVA for face area did not show any difference between emotional conditions. This was because the unpleasant and pleasant conditions contained more pictures depicting human faces photographed from long distances, whereas the images that contained faces in the neutral condition were mostly portraits taken from short distance. The ANOVA for saturation levels showed a slight effect between the stimulus categories [*F*_(2, 153)_= 3.15, *p* = 0.046], but *post-hoc* comparisons revealed no differences between the single image categories.

We also computed a saliency map for each four-image combination using the Saliency Toolbox (Walther and Koch, 2006) to further control for the possible bottom-up saliency effects in the parallel condition. A dyadic Gaussian pyramid was used for subsampling and three iterations were run for normalization. From the resulting saliency map the most salient location was extracted. In 21% of unpleasant trials, the emotional target was the most salient image, and in 23% of the pleasant trials, the target was the most salient image. A one-way ANOVA revealed no differences (*p* > 0.10) in the percentages of the most salient target images between the two conditions. These analyses suggested that low-level saliency could explain the attention effects to emotional targets in less than a chance level.

### Procedure

**Figure 1:**
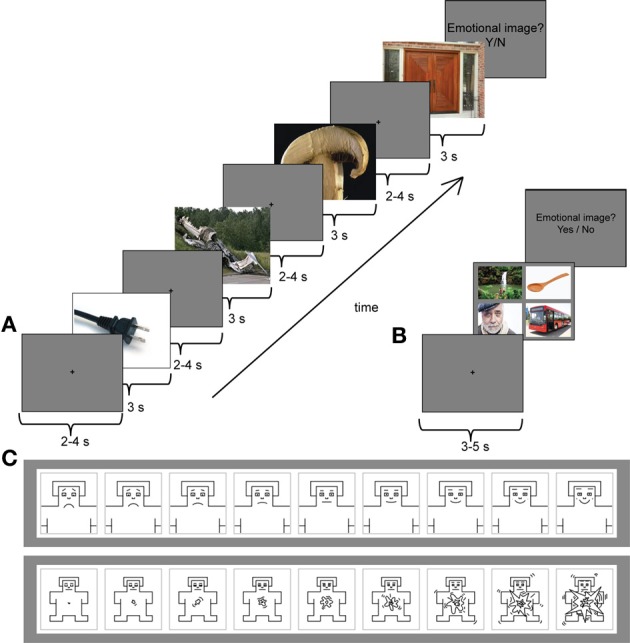
**(A)** An example trial from the serial viewing condition with an unpleasant target (plane crash) as the second image of the sequence. Each image was presented for 3 s followed by a central fixation cross on a gray background for 2–4 s (only one fixation cross is shown in this image). **(B)** A stimulus sequence from the parallel (free viewing) condition with a pleasant target image (people around waterfall) in the upper left corner. Participants had an unconstrained viewing of the stimulus images during which their eye movements were tracked. Before each stimulus set a central fixation cross was presented for 3–5 s. **(C)** The 9-point self-assessment manikin (SAM) scales were used to evaluate the emotional valence (upper panel) and arousal (lower panel) of the selected target image. ^*^Note that none of the example images are part of the experimental stimulus material.

Figure 1A presents the trial structure in the serial condition. Each image was presented for 3 s, followed by a central fixation cross, presented on a gray background for 2–4 s. The task of the participants was to view the images. After each trial, they were asked to indicate by “yes”/“no”—responses whether they detected an emotional image among the four images. If they clicked “yes,” the same four images were presented simultaneously on the screen, and the participants were asked to indicate the emotional image by a mouse-click. Subsequently, they were asked to rate the selected image with two 9-point scales (Figure 1C). The first scale measured the valence of the scenes from very unhappy (1) to very happy (9). The second scale measured the arousal of the scenes from very calm (1) to very excited (9). The emotional image appeared equally often, but randomly, as the first, second, third or fourth image of the trial. The trial length was approximately 20–30 s. In the serial condition, 40 pleasant, 40 unpleasant, and 4 neutral trials were presented. The number of neutral trials was kept intentionally low, because otherwise the experiment would have been unnecessarily long. For the ERP analyses in the serial condition, the images for neutral condition were selected randomly among images that preceded the emotional targets in the serial trials.

The parallel condition consisted of 40 pleasant, 40 unpleasant and 40 neutral trials (Figure 1B). Participants were instructed to look through all four images in a trial freely and to respond by clicking a mouse when they were ready to continue onto the next trial. The trial length in the parallel condition, thus, varied. Participants' eye movements were recorded only in the parallel condition. Between each four-image set a central fixation cross was presented for 3–5 s. After the presentation of the images, participants were asked to indicate whether they saw an emotional image among the four image-set. Similar to the serial condition, if they answered “yes,” the same set was presented again. The participants were then asked to indicate the selected image by a mouse-click and to rate that image using the valence and arousal scales. The serial and parallel conditions were presented in two blocks of each condition. The order of the blocks was counterbalanced across the participants.

### Data acquisition

During recordings, participants were comfortably seated in an electrically shielded room, and the stimuli were presented on a 22-inch display. EEG signal was recorded from 64 scalp sites using an elastic cap by BioSemi (BioSemi Inc., Amsterdam, The Netherlands) with the BioSemi ABC position system montage of Ag-AgCl active electrodes. Additionally, three active electrodes were placed at tip of the nose and at left and right mastoids. Blinks and eye movements were monitored by two bipolar leads. The electrodes were connected to a BioSemi ActiveTwo EEG amplifier. EEG data were recorded using BioSemi ActiView, and the signal was amplified and digitized at a rate of 2048 Hz.

In the parallel presentation condition, participants' eye movements were recorded concurrently with the EEG recordings, using a remote iView X™ RED250 (SensoMotoricInstruments, SMI, Teltow/Berlin, Germany) eye tracker. Positions of both eyes were sampled with 250 Hz from a viewing distance of 60 cm. Before each block, a 9-point calibration was performed. Eye movement and EEG recordings were synchronized with the stimulus sequence by Presentation™ software (Neurobehavioral Systems Inc., Albany, CA, USA). Accurate hardware synchronization is a basic requirement for the analysis of eye movement related brain potentials, since the latency and location of the gaze data is the basis of segmenting the ERP responses. In the current setup, the Presentation software was programmed to send a shared pulse to both datasets every few seconds. This ensured that the time points in the eye movement data corresponded with the EEG data.

### Eye movement analysis

Fixations and saccades were extracted from the raw eye coordinate data using an adaptive saccade detection algorithm (Nyström and Holmqvist, 2010). The initial parameters given to the algorithm were: velocity threshold of 100°/s, a minimum duration of 10 ms for saccade detection and minimum duration of 40 ms for fixation detection.

The eye movement data were analyzed with a 3 × 4 repeated-measures ANOVA with the emotional condition (pleasant, unpleasant, neutral) and the four quadrants in which the image was presented as within-participants factors. In order to preclude the possibility that parafoveal processing of emotional content would affect the processing of neutral images in the parallel condition, the neutral condition comprised of random images selected from the neutral trials. Early orienting of attention to images was measured as the target entry times and as the number of fixations before the first target entry from the stimulus onset. In addition, engagement of attention was measured as the number of fixations and the total dwell times (sum of fixation durations including re-fixations) per image. Moreover, we compared the likelihood of launching the first saccade toward the target image and the initial saccade latency between the emotional conditions.

### Control analysis for oculomotor factors

A critical factor in the analysis of eye movement related brain potentials is to control for the systematic differences in oculomotor variables (e.g., saccade amplitudes and fixation durations), since systematic differences in eye movement measures are coupled with changes in the phases of the overlapping potentials (Dimigen et al., 2011). Previous research has also shown that saccade kinematics influences the amplitudes and waveforms of the eye fixation related potentials, EFRPs. That is, the amplitude of the spike potential (SP, an electrical eye muscle activity at saccade onset) increases with saccade size (see Keren et al., 2010; Dimigen et al., 2011). Moreover, the amplitudes of incoming saccades have been shown to influence the Lambda response, a response elicited by the afferent information inflow at the beginning of a fixation (Kazai and Yagi, 2003). The SP amplitudes gradually diminish from extra-ocular channels toward posterior sites. However, their scalp topography is strongly modulated by the direction of saccades with the scalp distributions biased toward the saccade direction. Therefore, the influences caused by differences in eye movement patterns need to be controlled in the EFRP analyses. We calculated the directions, amplitudes, and durations of pre-target saccades across the emotional conditions in the parallel condition (Table 2). In the stimulus onset-locked averaging of ERPs (in the serial condition), the effect of SP is nearly eliminated due to the latency jitter of the biphasic deflections. In order to control for the possible artifacts caused by within-image saccadic eye movements, we also calculated the number of within-image saccades and their amplitudes and directions in the 500 ms time window that was critical for the ERP-analysis in the parallel condition (Table 2).

**Table 2:** **Means and (standard deviations) of the affective ratings and eye movement measures across the emotional conditions**.

	**Pleasant pictures**	**Unpleasant pictures**	**Neutral pictures**
Valence ratings	6.72 (0.34)	2.39 (0.51)	
Arousal ratings	3.78 (1.40)	5.29 (1.47)	
Task duration (s)	7.43 (2.58)	7.91 (2.52)	6.36 (3.20)
First saccade to target (%)	37.12 (7.23)	46.00 (15.07)	23.15 (11.50)
Initial saccade latency (s)	0.52 (0.26)	0.63 (0.42)	0.57 (0.26)
Target entry time (s)	1.38 (0.95)	1.19 (0.81)	1.77 (0.85)
Dwell time (s)	0.72 (0.16)	0.76 (0.20)	0.55 (0.22)
No. fix before target	2.60 (0.57)	2.17 (0.59)	3.27 (0.49)
No. fix on target	4.50 (1.94)	5.87 (2.76)	2.50 (1.17)
**INCOMING SACCADES**			
Amplitude (deg)	12.92 (1.42)	11.94 (1.66)	13.43 (2.12)
Angle (deg)	173.09 (17.11)	171.09 (24.06)	176.05 (17.77)
Duration (ms)	65.46 (8.70)	61.22 (9.20)	65.72 (11.19)
**WITHIN-TARGET IMAGE SACCADES IN THE 0–500 MS TIME WINDOW**			
Amplitude (deg)	6.53 (2.51)	6.39 (3.15)	10.86 (5.21)
Angle (deg)	186.86 (29.17)	182.25 (37.69)	157.25 (34.59)
Count	1.51 (0.25)	1.40 (0.18)	1.53 (0.20)

Moreover, to further control for the possible associated effects between eye movement variables and the amplitudes of the brain potentials, parafoveal processing of emotional stimuli was examined at a single-trial level. This was done by including pre-target saccade amplitudes and first target-fixation durations as covariates to a (conditional) liner mixed model (Hox, 2002, implemented in SPSS Version 20 Mac, IBM Corporation, New York, United States) considering the single-trial ERP amplitudes selected around the first target entry.

### EEG analyses

EEG data were processed with BESA (Version. 5.2; MEGIS Software, Graefelfing, Germany). Amplified voltages originally referenced to nose were rereferenced offline to linked mastoids, resampled to 512 Hz and off-line filtered with 0.5–40 Hz band pass.

The fluctuating electrical fields produced by eyelid movements and the rotation of the eyeball's corneoretinal dipole propagate to EEG electrodes and contaminate the recording of brain activity (Berg and Scherg, 1991; Rugg and Coles, 1995; Plöchl et al., 2012). Ocular artifacts make the analysis of eye movement related brain potentials challenging. One way to avoid the ocular artifacts is to restrict the EFRP analyses to the fixation period when the eye is relatively still (Baccino and Manunta, 2005; Simola et al., 2009). When the analysis is restricted only to the fixation period, it is possible to analyze the early sensory ERP components such as the P1/N1 or P2/N2 components (Olofsson et al., 2008). However, because the oculomotor and cognitive systems are partly independent, the eyes can leave the target before processing is completed (see Kliegl et al., 2012) and as a result, some events of interest occur at latencies that exceed the fixation duration. For example, in reading there is a discrepancy between the typical fixation durations (200–250 ms) and the latency of the N400 component, a robust measure of semantic processing that peaks around 400 ms post-stimulus (Kutas and Hillyard, 1980). Thus, in normal reading situations, the eyes have already left the word when the N400 related to that word peaks. Despite this fact, the N400 has been successfully studied during a normal reading by using the EFRP analysis method (Dimigen et al., 2011; Kliegl et al., 2012). Such conditions require careful ocular artifact correction that spares the genuine brain activity. In the present study, corneoretinal eye movement artifacts were corrected using a principal component analysis (PCA)-based spatial filter (Ille et al., 2002). In order not to remove brain activity related to the stimulus processing (see Dimigen et al., 2011), we defined the representative PCA components for eye blink and eye movement artifacts manually outside the experimental trials^1^. Other remaining artifacts were removed automatically with ±160 μ V rejection level.

In the serial condition, the EEG signal was time-locked to the stimulus onset and segmented into epochs extending from −200 to 1500 ms around stimulus onset. The epochs were baseline corrected relative to 100 ms pre-stimulus interval. In the parallel condition, the EEG data were time-locked to the point at which the eyes first entered an emotional image in pleasant and unpleasant trials or a randomly selected image in the neutral trials. The EEG was segmented into epochs from −200 to 1500 ms that were baseline corrected relative to −200 to −100 ms interval before the first eye entry to the target image. The baseline correction was performed before the saccade onsets in order to avoid temporal overlap with the saccadic spike potentials. Moreover, to investigate the time-course of parafoveal processing of emotional stimuli, the ERP responses in the parallel condition were also time-locked to the stimulus onset and segmented into epochs of −200 to 1500 ms with 100 ms pre-stimulus baseline. In both conditions, the data were averaged according to the emotional condition: pleasant, unpleasant, or neutral.

The time windows for the ERP analyses were selected based on visually detected components. In the serial condition, modulation by emotional content was detected at 80–120 ms and at 220–280 ms time windows. In the parallel condition, a positive component at 125–175 ms was observed. In addition, a later sustained positive response, most likely the LPP response, was observed for both presentation conditions. An ANOVA for the LPP peak latencies revealed no differences between the presentation conditions [*F*_(1, 10)_= 2.81, *p* = ns.] (serial condition: 387.65 ± 38.12 SD; parallel condition: 362.15 ± 41.86 SD). Because the response was sustained, the mean amplitudes were calculated in the 400–500 ms time window. In the serial condition, 7% of the trials were excluded based on the automatic artifact rejection (±160 μ V) criteria. The number of trials that entered the ERP-analysis by emotional conditions were: pleasant: 37.1; unpleasant: 37.0; neutral: 37.5. In the parallel condition, 2% of the trials were excluded because the detection of image entry from the eye movement data failed. An additional 7% of the trials were excluded based on the artifact rejection criteria. The number of trials accepted in the parallel condition were: pleasant: 37.0; unpleasant: 36.8; neutral: 35.9. The mean amplitudes were calculated for nine electrodes along the anterior-posterior axis: anterior (F3, Fz, F4), central (C3, Cz, C4) and posterior (P3, Pz, P4), and into three hemispheric groups: left (F3, C3, P3), midline (Fz, Cz, Pz), and right (F4, C4, P4). The mean amplitudes of LPPs were subjected to 2 × 3 × 3 × 3 repeated measures ANOVA with the factors: presentation condition (serial, parallel), emotional condition (pleasant, unpleasant, neutral), anterior-posterior axis (anterior, central, posterior), and laterality (left, midline, right). The mean ERP amplitudes at 80–120 ms and at 220–280 ms time windows in the serial condition and at 125–175 ms in the parallel condition were analyzed with 3 × 3 × 3 repeated measures ANOVA with the following factors: emotional condition (pleasant, unpleasant, neutral), anterior-posterior axis (anterior, central, posterior), and laterality (left, midline, right). *Post-hoc* multiple comparisons were Bonferroni corrected, and the *p*-values were corrected according to the Greenhouse-Geisser procedure when the sphericity assumption was violated.

## Results

### Behavioral results

Affective ratings confirmed the differences between emotional image contents (Table 2). Pleasant images were judged as more pleasant than unpleasant images [*F*_(1, 0)_= 357.44, *p* < 0.001, η^2^_*p*_ = 0.97], and the unpleasant pictures were rated higher on arousal than the pleasant pictures [*F*_(1, 10)_= 14.54, *p* = 0.003, η^2^_*p*_ = 0.59]. In the parallel condition, the quadrant in which the image was presented did not affect the valence or arousal ratings. Moreover, the trial durations in the parallel condition did not differ between emotional conditions or between the quadrants in which the emotional image was presented. The focus of this study was on the effects of emotional valence rather than on emotional arousal. Therefore, the following analyses are only performed for the three different emotional valence categories.

### Eye movement results

#### Orienting of attention

The likelihood of launching *the first saccade to target* differed across the conditions [*F*_(2, 20)_= 13.17, *p* < 0.001, η^2^_*p*_ = 0.579] with higher likelihood of launching the first saccade toward unpleasant [*t*_(9)_ = 4.32, *p* = 0.005] and pleasant [*t*_(9)_ = 3.41, *p* = 0.019] than toward neutral target images. There were no differences in the likelihood of launching the first saccade to pleasant or unpleasant targets. *The first saccade latencies* did not differ between the emotional conditions. *The target entry times* were affected by the emotional conditions [*F*_(2, 18)_ = 12.91, *p* < 0.001, η^2^_*p*_ = 0.59], indicating that unpleasant [*t*_(9)_ = −4.70, *p* = 0.003] and pleasant [*t*_(9)_ = −3.51, *p* = 0.020] images were entered earlier than neutral images (Table 2). However, no differences occurred between the entry times to unpleasant and pleasant images. A main effect of location was also observed [*F*_(1.38, 12.43)_= 4.37, *p* = 0.048, η^2^_*p*_ = 0.33], suggesting that images in the upper right [*t*_(9)_ = 3.76, *p* = 0.027] quadrant were entered earlier than images in the lower right quadrant. The interaction between emotional condition and image location was not significant. *The number of fixations before* the target image was entered for the first time varied also between the emotional conditions [*F*_(2, 18)_ = 18.96, *p* < 0.001, η^2^_*p*_= 0.68] and between quadrants [*F*_(3, 27)_ = 9.49, *p* < 0.001, η^2^_*p*_ = 0.51]. These results showed that pleasant [*t*_(9)_ = −3.41, *p* = 0.008] and unpleasant [*t*_(9)_ = 5.27, *p* = 0.002] images were fixated earlier than neutral images, and that unpleasant images were fixated earlier than pleasant images [*t*_(9)_ = 3.51, *p* = 0.020]. The images at lower right quadrant were fixated later than images at other quadrants.

#### Engagement of attention

The emotional conditions differed also in *the number of fixations* on an image [*F*_(1.18, 10.61)_= 32.85, *p* < 0.001, η^2^_*p*_ = 0.79]. That is, unpleasant [*t*_(9)_ = 5.96, *p* = 0.001] and pleasant [*t*_(9)_ = 6.82, *p* < 0.001] images were fixated more often than neutral images, and unpleasant images were fixated more often than pleasant images [*t*_(9)_ = −3.96, *p* = 0.010]. Image location did not affect the amount of fixations on an image. *The dwell times* (i.e., the sum of fixation durations) showed differences between the emotional conditions [*F*_(1.24, 11.16)_= 5.46, *p* = 0.034, η^2^_*p*_ = 0.38]. That is, pleasant [*t*_(9)_ = 3.65, *p* = 0.016] images were looked at longer than neutral images. The image location did not affect the dwell times.

#### Saccade kinematics

The control analysis of saccade kinematics suggested no differences between emotional conditions in terms of directions of incoming saccades from the target (Table 2). The incoming saccade amplitudes did not differ between emotional conditions, but the incoming saccade durations differed [*F*_(2, 18)_ = 3.93, *p* = 0.041, η^2^_*p*_ = 0.30] with shorter saccade durations before unpleasant than before pleasant targets [*t*_(9)_ = 2.96, *p* = 0.048].

In the parallel condition, the ERPs were time-locked to the first target entry and were not restricted to fixation period. To control for the effects of within-image saccadic eye movements, we calculated the number of within-image saccades and their amplitudes and directions in the 500 ms time window that was critical for the ERP-analysis (Table 2). This analysis revealed that emotional conditions differed in the within-image saccade amplitudes [*F*_(2,18)_ = 13.58, *p* < 0.001, η^2^_*p*_ = 0.60], suggesting more widespread saccades in the neutral condition as compared to the unpleasant [*t*_(9)_ = 4.15, *p* = 0.003] and pleasant [*t*_(9)_ = 3.75, *p* = 0.014] conditions. Neither the number of within-image saccades nor their directions differed between the emotional conditions.

### EEG results

#### Eye movement related potentials

**Figure 2:**
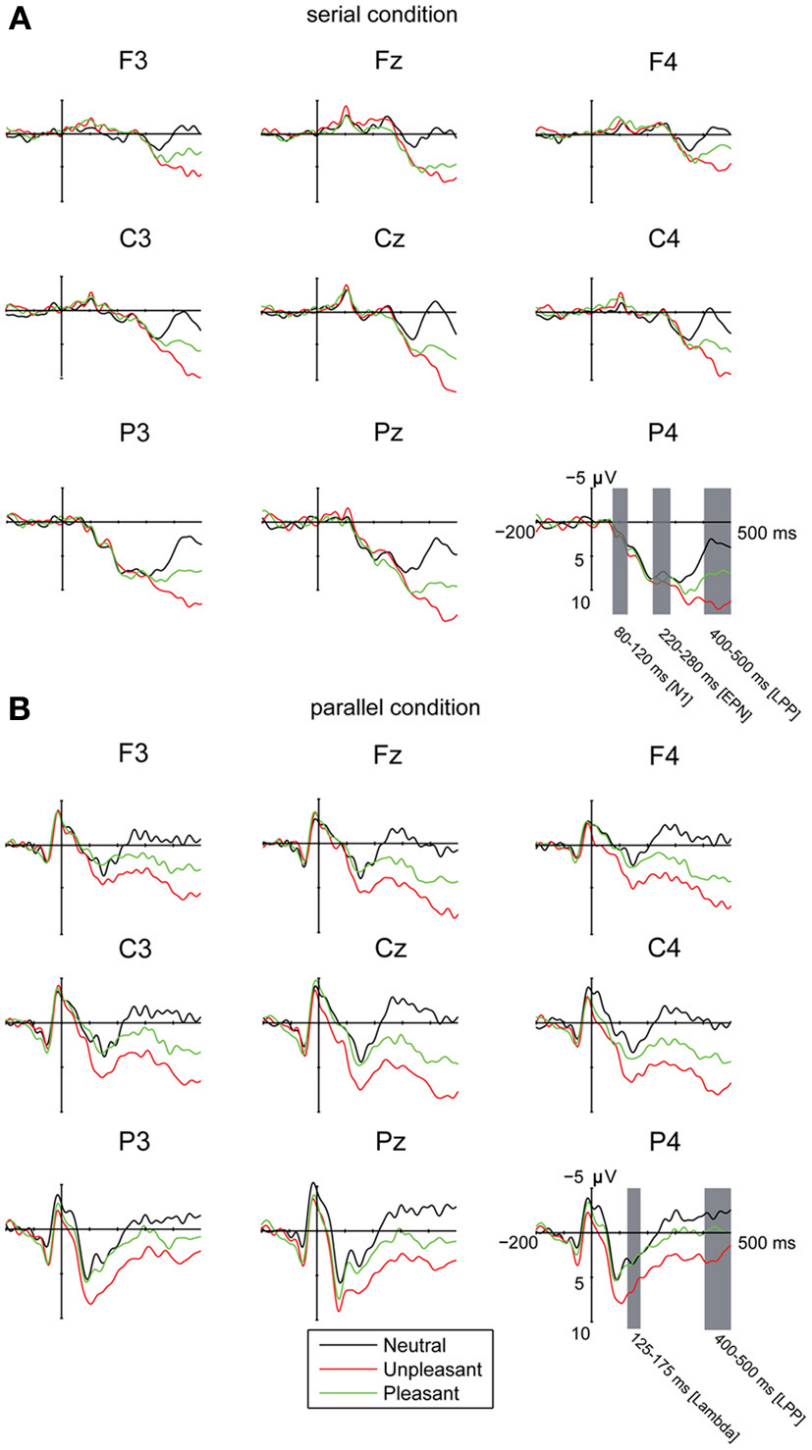
**Grand averages of ERPs (A) in the serial visual presentation when the ERPs were time-locked to stimulus onset, and (B) in the parallel condition, when the ERPs were time-locked to the first eye entry to a target image.** A 30 Hz filter was used for data plotting.

In order to control for the possibility that the emotional LPP-responses in the parallel condition were affected by earlier differences between the emotional conditions during or after the offset of the saccadic eye movement, we analyzed the ERP amplitudes between −50 and 50 ms around the first target entry (i.e., at the time-locking point). The analysis revealed a three-way interaction of emotional condition × laterality × electrode position [*F*_(8, 80)_ = 2.22, *p* = 0.034, η^2^_*p*_ = 0.18]. The *post-hoc* analyses suggested only minor differences in the response topographies between the emotional conditions. The responses for the unpleasant images over the left hemisphere were more negative at anterior [*t*_(9)_ = 3.14, *p* = 0.032] and central [*t*_(9)_ = 3.70, *p* = 0.012] than at posterior electrode sites. Moreover, the responses at posterior sites in the unpleasant condition were more negative over midline than left hemisphere [*t*_(9)_ = 3.41, *p* = 0.020]. In the pleasant condition, the central responses were more negative over midline than at right hemisphere [*t*_(9)_ = 3.45, *p* = 0.019]. The differences in emotional conditions around the time-locking point (Figure 2B) did not account for the much larger and systematic differences between emotional conditions that were observed at 400–500 ms after target entry.

The EFRPs locked to the first target entry showed a positive response in the time window of 125–175 ms. This component was emphasized in the central and parietal electrode sites (Figure 2B). The analyses showed no differences in the peak amplitude latencies or amplitudes between the emotional conditions, laterality or in the anterior-posterior axis. This component is most likely the Lambda response, which occurs as a response to the afferent information inflow at the beginning of a fixation (Kazai and Yagi, 2003). In the present experiment, the Lambda responses were smeared and peaked relatively late (at 150 ms) because the responses were not time-locked to the fixation onset but to the time point when the eyes crossed the border of the target image. The eyes were, thus, still moving at the time-locking point. Most likely due to differences in saccade durations, there is some jitter in the latencies of the Lambda responses resulting in longer responses than the ones typically observed in studies using the co-registration of eye movements and EEG.

#### Emotional response

In order to investigate the brain responses to emotional images, the LPP was analyzed in the time window of 400–500 ms for both presentation conditions (Figures 2A,B, Table 3). The results showed that responses were larger during serial presentation than during parallel presentation of images [*F*_(1,10)_ = 8.42, *p* = 0.016, η^2^_*p*_ = 46]. Further, these analyses showed that the LPPs differed between the emotional conditions [*F*_(2,20)_ = 63.07, *p* = 0.001, η^2^_*p*_ = 0.86], suggesting stronger responses for unpleasant [*t*_(9)_ = −9.76, *p* < 0.001] and pleasant [*t*_(9)_= −6.67, *p* < 0.001] images than for the neutral images. Moreover, the responses were stronger for unpleasant than for pleasant images [*t*_(9)_ = 5.60, *p* = 0.001]. The LPPs differed also along the anterior posterior axis [*F*_(2, 20)_ = 6.33, *p* = 0.021, η^2^_*p*_ = 0.39]. Overall, the LPP responses were stronger on the central than on the anterior electrode sites [*t*_(9)_= −5.97, *p* < 0.001]. The results also showed a main effect of laterality [*F*_(2, 20)_ = 5.96, *p* = 0.012, η^2^_*p*_ = 0.37] with stronger responses on the midline than over the right hemisphere [*t*_(9)_= 3.36, *p* = 0.022). The main effects were modulated by an interactions between the emotional condition × laterality [*F*_(4,40)_ = 6.23, *p* = 0.001, η^2^_*p*_ = 0.38], suggesting that for unpleasant and pleasant conditions the responses were stronger over the midline than the left [unpleasant: *t*_(9)_ = −3.36. *p* = 0.022; pleasant: *t*_(9)_ = −2.89, *p* = 0.048] or the right hemisphere [unpleasant: *t*_(9)_ = 4.52, *p* = 0.003; pleasant: *t*_(9)_ = 3.50, *p* = 0.017].

**Table 3:** **Mean amplitudes and peak latencies of the LPP response (400–500 ms) across the studied electrode sites for the presentation conditions (Serial, Parallel) and for each emotional condition (Unpleasant, Pleasant, Neutral)**.

**Mean amplitude (μV ± SD)**								
**Mean latency of peak (ms ± SD)**								
**F3**	**Fz**	**F4**	**C3**	**Cz**	**C4**	**P3**	**Pz**	**P4**
**SERIAL**								
**Unpleasant**								
5.16 ± 2.26	5.99 ± 2.99	4.38 ± 3.02	8.14 ± 3.89	9.20 ± 4.03	7.49 ± 3.95	10.96 ± 4.46	12.66 ± 4.45	11.27 ± 4.24
427.20 ± 50.61	433.42 ± 47.77	405.72 ± 77.00	439.63 ± 47.62	457.03 ± 43.56	448.69 ± 46.56	448.33 ± 46.80	427.20 ± 54.63	409.98 ± 77.08
**Pleasant**								
2.61 ± 3.58	4.37 ± 4.64	1.94 ± 3.56	4.68 ± 3.42	5.26 ± 3.61	4.48 ± 2.64	6.75 ± 2.21	8.55 ± 3.38	7.02 ± 2.82
412.11 ± 40.98	410.87 ± 54.67	364.17 ± 94.71	403.41 ± 92.30	411.04 ± 75.23	426.31 ± 59.83	330.25 ± 84.90	364.17 ± 94.71	336.11 ± 80.94
**Neutral**								
−0.72 ± 1.83	−0.38 ± 2.55	−0.59 ± 2.22	0.99 ± 2.50	−0.02 ± 2.90	0.87 ± 2.42	2.54 ± 3.03	3.21 ± 2.81	3.00 ± 2.52
354.23 ± 80.69	382.64 ± 76.00	366.48 ± 69.99	362.92 ± 67.25	368.96 ± 84.52	372.16 ± 84.75	291.37 ± 65.48	316.40 ± 89.48	295.45 ± 72.71
**PARALLEL**								
**Unpleasant**								
5.35 ± 3.32	7.25 ± 3.80	6.10 ± 3.76	6.15 ± 3.62	7.67 ± 3.56	7.05 ± 3.58	3.15 ± 4.12	3.73 ± 3.91	2.63 ± 4.00
394.88 ± 77.58	405.54 ± 87.72	438.39 ± 48.80	407.31 ± 84.00	428.09 ± 89.60	422.05 ± 66.54	328.48 ± 103.88	371.80 ± 87.47	328.30 ± 107.30
**Pleasant**								
2.36 ± 2.38	3.76 ± 1.34	3.36 ± 2.06	2.78 ± 1.81	3.83 ± 2.23	3.64 ± 1.37	0.89 ± 3.08	0.99 ± 3.28	−0.33 ± 3.68
386.19 ± 79.18	400.21 ± 65.52	406.60 ± 53.50	367.54 ± 89.54	390.80 ± 102.81	398.44 ± 64.32	343.04 ± 89.01	352.63 ± 102.31	321.91 ± 106.07
**Neutral**								
−0.65 ± 2.64	0.54 ± 2.73	−0.65 ± 2.46	−0.75 ± 2.19	−0.64 ± 2.65	0.03 ± 2.61	−1.36 ± 3.39	−2.42 ± 2.93	−2.04 ± 2.33
317.65 ± 87.86	371.98 ± 121.10	373.93 ± 85.77	351.39 ± 111.66	305.98 ± 116.71	354.05 ± 101.28	284.80 ± 108.46	271.31 ± 101.73	256.04 ± 67.31

**Figure 3:**
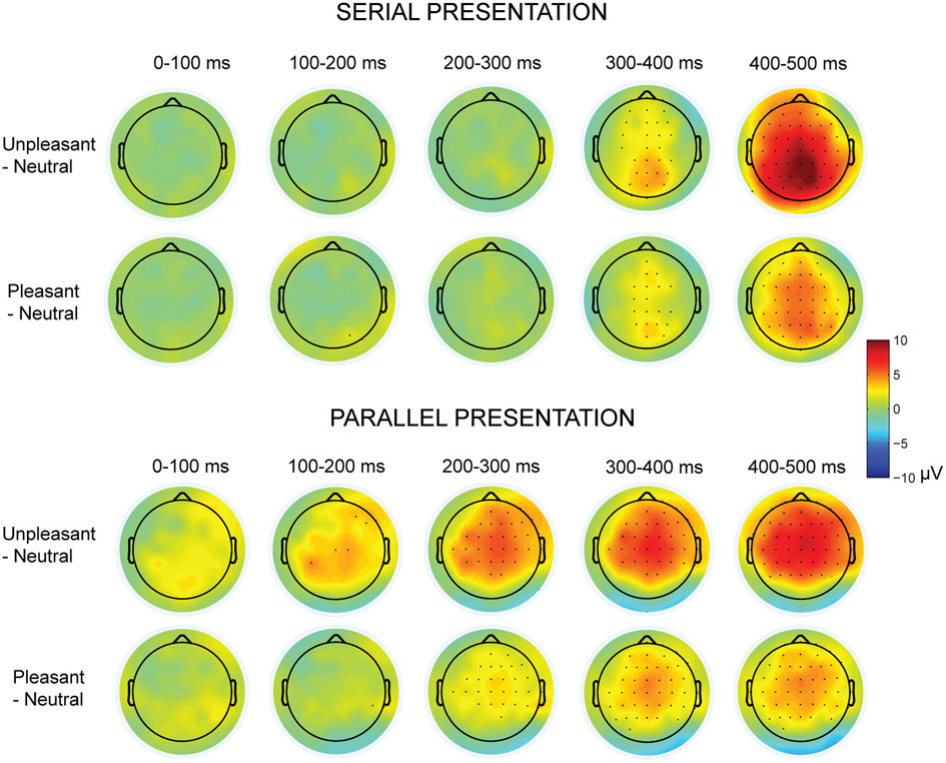
**Topographic maps displaying the scalp distributions of the differences between emotional–neutral conditions.** The top rows show the differences between emotional and neutral conditions in the serial visual presentation. The bottom rows show the scalp distributions in the parallel presentation, when the responses were time-locked to the first target entry. Marked channels depict a significant (*p* < 0.05) difference in one-sample *t*-test.

**Figure 4:**
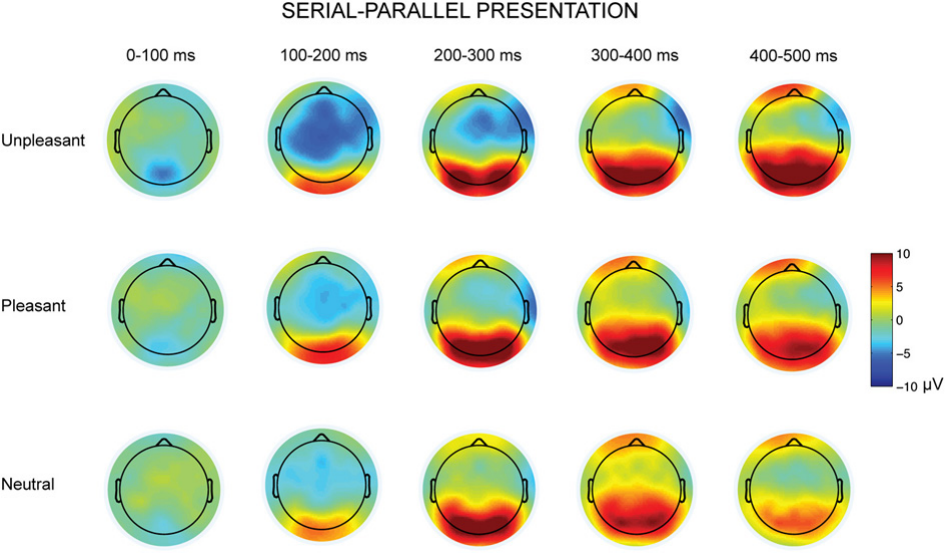
**Topographic maps displaying the scalp distributions of the differences between serial–parallel conditions for unpleasant (top row), pleasant (middle row) and neutral condition (bottom row)**.

The parallel and serial presentation conditions differed also in response topographies (Figures 3, 4). This was indicated by the interaction between the presentation condition × emotional condition × anterior-posterior axis [*F*_(4, 40)_ = 10.03, *p* = 0.001, η^2^_*p*_ = 0.50], which suggested that for all conditions the parietal responses were larger during serial than during parallel presentation [unpleasant: *t*_(9)_ = −4.15, *p* = 0.002, pleasant: *t*_(9)_ = −5.38, *p* < 0.001, neutral: *t*_(9)_ = −3.68, *p* = 0.004) (Figure 4). Further, the interaction between the presentation condition × laterality × anterior-posterior axis [*F*_(4,40)_ = 3.91, *p* = 0.009, η^2^_*p*_ = 0.28] showed that during serial presentation, the responses were enhanced across all parietal sites (left: *t*_(9)_ = −3.70, *p* = 0.004, midline: *t*_(9)_ = −4.99, *p* = 0.001, right: *t*_(9)_ = −5.39, *p* < 0.001) as compared to the parallel presentation. In the parallel condition, the responses were stronger over the frontal midline than over the frontal left site [*t*_(9)_ = 4.03, *p* = 0.007]. In the serial condition, the frontal responses were stronger over midline than over the right site [*t*_(9)_ = 3.14, *p* = 0.011]. Moreover, in the parallel condition, the responses were enhanced at frontal [midline: *t*_(9)_ = 4.83, *p* = 0.002, right: *t*_(9)_ = 3.21, *p* = 0.009] and central [midline: *t*_(9)_ = 6.16, *p* < 0.001, right: *t*_(9)_= 4.86, *p* = 0.001] sites as compared to the parietal sites. In contrast, during serial presentation, the responses were stronger over central than over the frontal sites [eft: *t*_(9)_ = 3.41, *p* = 0.020, right: *t*_(9)_ = 4.02, *p* = 0.007]. Further, the responses in the serial condition were enhanced at parietal as compared to the central sites [left: *t*_(9)_ = 3.06, *p* = 0.036; midline: *t*_(9)_ = 6.72, *p* < 0.001].

The presentation condition did not affect the LPP peak amplitude latencies. However, latencies of the LPP peak responses (between 200 and 500 ms time window) differed between emotional conditions [*F*_(2, 20)_= 19.56, *p* < 0.001, η^2^_*p*_ = 0.66], suggesting that the responses for unpleasant [*t*_(9)_ = 6.44, *p* < 0.001] and pleasant [*t*_(9)_ = 3.07, *p* = 0.036] images peaked later than the responses for neutral images. Also the unpleasant responses peaked later than the pleasant responses [*t*_(9)_ = 3.18, *p* = 0.030]. Further, the LPP peak response latencies differed along the anterior-posterior axis [*F*_(2, 20)_ = 26.40, *p* < 0.001, η^2^_*p*_ = 0.73] with earlier peak responses at the parietal than at the central [*t*_(9)_ = 6.76, *p* < 0.001] or the frontal [*t*_(9)_ = 6.41, *p* = 0.001] electrode sites. Further, we performed one-sample *t*-tests for all recorded EEG channels to test whether the subtraction curves between emotional and neutral conditions differed from zero (Figure 3). These analyses suggested that the responses to emotional (vs. neutral) images started to deviate earlier (around 100 ms) in the parallel than in the serial condition.

#### Parafoveal processing of emotional content

**Figure 5:**
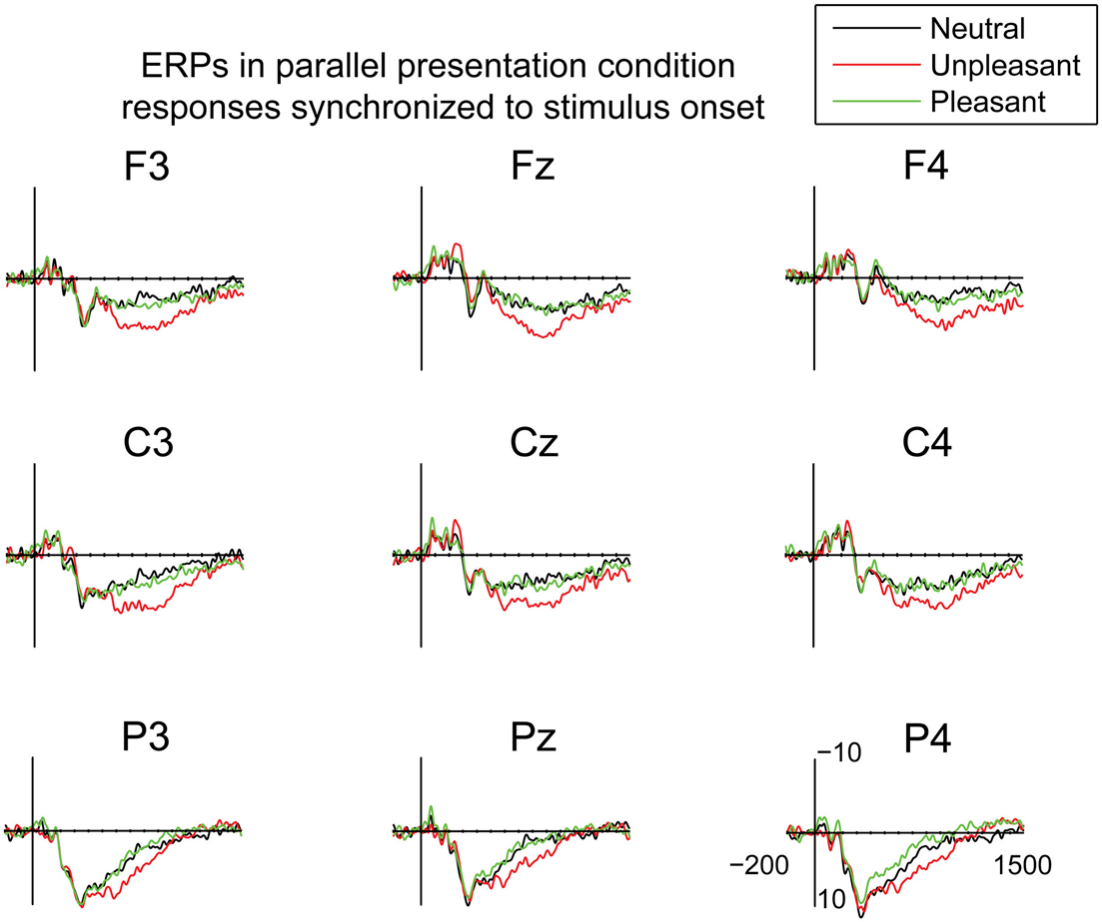
**Grand average ERPs time-locked to the stimulus onset in the parallel condition.** A 30 Hz filter was used for data plotting.

In order to examine the early attentional orienting to emotional scenes, the ERPs in the parallel condition were also time-locked to the stimulus onset (Figure 5). The mean amplitudes of these responses were analyzed in 100 ms bins between 0 and 700 ms post-stimulus. In the time-window of 0–100 ms, the analyses showed a difference between emotional conditions [*F*_(2, 20)_ = 5.11, *p* = 0.016, η^2^_*p*_ = 0.34], suggesting more negative responses for the pleasant as compared to the unpleasant images [*t*_(9)_ = 2.90, *p* = 0.047]. The responses at 100–200 ms, 200–300 ms, 300–400 ms, 400–500, and 500–600 ms did not reveal any differences between the emotional conditions. At 600–700 post-stimulus, a main effect of emotional condition occurred [*F*_(2, 20)_ = 3.67, *p* = 0.044, η^2^_*p*_ = 0.27], suggesting numerically larger positive deflections for unpleasant than for neutral scenes. The responses to unpleasant scenes were also larger than the responses to pleasant scenes, but these differences did not reach significance in the *post-hoc* multiple comparisons. The results, thus, showed that emotional stimulus content did not modulate the ERP responses until around 600 ms after stimulus onset in the parallel condition.

To further examine the parafoveal processing of emotional content, the ERP- amplitudes in the parallel condition were examined at a single-trial level. This was done by a linear mixed model, which considered the pre-target saccade amplitudes and the first target-fixation durations as covariates for the single-trial ERP amplitudes. The analysis revealed no relationship between the pre-target saccade amplitudes and ERP-responses at 125–175 ms and 400–500 ms time windows, suggesting that the distance from which saccades were launched toward the target images did not affect the ERP amplitudes. Thus, these analyses supported no parafoveal processing of emotional content. Further, the analysis controlled for the possible associated effects between eye movement variables and ERP-responses, by showing that the ERP amplitudes at 125–175 and 400–500 ms were not modulated by systematic differences in first target-fixation durations.

#### Early modulation of responses in the serial condition

Visual inspection of the waveforms in the serial condition revealed a negative (N1) response at 80–120 ms (Figure 2). Laterality affected these responses [*F*_(2, 20)_= 10.32, *p* = 0.001, η^2^_*p*_ = 0.51], suggesting enhanced negative responses at midline than at right electrode sites [*t*_(9)_ = 4.43, *p* = 0.004]. Moreover, the N1 responses differed along the anterior posterior axis [*F*_(2, 20)_ = 17.46, *p* = 0.001, η^2^_*p*_ = 0.64], suggesting enhanced negative response at frontal [*t*_(9)_ = 3.86, *p* = 0.010] and central [*t*_(9)_ = 4.86, *p* = 0.002] as compared to parietal sites.

Additionally, the waveforms in the serial condition contained a negative going wave at 220–280 ms (Figure 2). The latency of this response corresponds to the timeline of the EPN response that is often found in studies of emotional processing (Olofsson et al., 2008). The analysis showed that the EPN amplitudes differed along the anterior posterior axis [*F*_(2, 20)_ = 40.40, *p* < 0.001, η^2^_*p*_ = 0.80], suggesting more negative responses at frontal as compared to the central [*t*_(9)_ = 7.81, *p* < 0.001] and parietal [*t*_(9)_= 6.50, *p* < 0.001] electrode sites. The analyses also revealed an interaction between laterality × anterior posterior axis [*F*_(4, 40)_ = 6.30, *p* < 0.001, η^2^_*p*_ = 0.39], suggesting more negative responses at frontal [left: *t*_(9)_ = 5.79, *p* < 0.001; midline: *t*_(9)_ = 5.25, *p* = 0.001; right: *t*_(9)_ = 07.03, *p* < 0.001] and central [left: *t*_(9)_ = 7.21, *p* < 0.001; midline: *t*_(9)_ = 5.84, *p* < 0.001; right: *t*_(9)_ = 8.48, *p* < 0.001] than at parietal sites. At parietal sites, the responses were more negative at midline than over the right hemisphere [*t*_(9)_ = 3.48, *p* = 0.018].

## Discussion

### Allocation of attention to emotional content during free viewing

The present study had two aims. The first aim was to investigate the time course of attention and emotion processes during free viewing of emotional scenes. Previous research has found no consensus on the role of attention on emotional processing. Some studies suggest that attention is automatically directed toward emotional stimuli (Öhman et al., 2001; Blanchette, 2006; Fox et al., 2007), while other researchers propose that emotional processing depends on attentional resources allocated to process the emotional content (Pessoa et al., 2002; Holmes et al., 2003). A third approach suggests a fast and involuntary attention capture by emotional content, which is sensitive to regulatory attentional influences (e.g., Calvo and Nummenmaa, 2007).

In the present study, co-registration of eye movement and EEG data was used to address the time course of attention to emotional stimuli during free viewing. The eye movement data supported previous research (Calvo and Lang, 2004; Nummenmaa et al., 2006, 2009; Coy and Hutton, 2012) in showing that viewers' attention was captured faster by emotional than by neutral content of the stimuli. This was indicated by earlier target entry times, decreased number of fixations before the target entry and higher likelihood of launching the first saccades toward the emotional than for the neutral scenes. Subsequently, sustained attentional focus on emotional stimuli was indexed in larger number of fixations and in longer dwell times for emotional than for neutral pictures. These results suggested that attention was engaged for a longer time, possibly in order to more fully process the emotional significance of the stimuli. The eye movement results, thus, showed that emotional images were detected faster in the parafoveal or peripheral visual fields, and were entered earlier with the eyes than neutral pictures. Previous research assumes that shifts of covert visual attention precede eye movements to a location in space (Deubel and Schneider, 1996). The finding that initial fixations occurred earlier to emotional than to neutral images implies that covert attention to emotional content was driving overt attention toward emotional content faster than to neutral content.

The ERP responses time-locked to the first target entry showed enlarged responses to both unpleasant and pleasant stimuli at 400–500 ms post target entry. The latency and topography of these responses correspond to the “late positive potential,” LPP, response. A long lasting elevated positivity when participants attend to emotional pictures is a well-established finding in emotional research (Olofsson et al., 2008). However, the responses time-locked to the stimulus display onset in the parallel condition suggested no differences between the emotional conditions until around 600 ms from the stimulus onset. This time-course corresponds with the eye movement data, indicating that participants made approximately two fixations before they entered the unpleasant image with their eyes. The ERP data, thus, did not support parafoveal processing of emotional stimuli. Furthermore, the single-trial analysis that combined eye movement and ERP measures to examine the effects of pre-target saccade amplitudes on the ERP responses showed no relationship between the eye movement and ERP measures. Although the ERP analysis supported no parafoveal preview effects, there was some indication that the emotional conditions began to differ from each other earlier in parallel than in serial viewing condition (Figures 2, 3). The one-sample *t*-tests performed for the difference curves between emotional and neutral conditions showed that the emotional responses occurred approximately 100 ms earlier in the parallel than in the serial condition. This could indicate a parafoveal preview effect (see Dimigen et al., 2011; Kliegl et al., 2012). However, the analysis supported no differences in the peak latencies for the LPP responses between the presentation conditions. Further, with the current setup, the latency differences in emotional responses cannot be dissociated from the temporal difference in baseline periods between the viewing conditions (also 100 ms earlier in the parallel condition).

Our results, thus, support the view according to which overt spatial attention needs to be directed to emotional content first before the ERP responses to emotional content could be observed. Similar findings have been previously reported in ERPs by Holmes et al. (2003) and by Pessoa et al. (2002) using fMRI. These findings suggest an involvement of higher-level processes in the interaction between emotion and attention. Moreover, both eye movement and EEG results demonstrated enhanced attention to emotional as compared to neutral scenes, supporting “the emotionality hypothesis.” The ERP and eye movement results further confirmed the “negativity hypothesis” (Ito et al., 1998; Smith et al., 2003; Hajcak and Olvet, 2008) by showing larger LPP responses to unpleasant than to pleasant stimuli and faster attention capture by the unpleasant than pleasant scenes in terms of the number of fixations made before the first target entry. The unpleasant scenes also engaged attention for a longer duration. This was indicated by a larger number of fixations on unpleasant than on pleasant images.

### Validation of the co-registration technique

The second aim was to validate the co-registration technique. In the EEG-analysis, the emotional effects were first established in the SVP, which provided a foundation to investigate the emotional processing during parallel presentation of images. In the parallel condition, the ERP responses were time-locked to the first target entry times. Previous research indicates that emotional scene content can be processed in the parafoveal or peripheral visual fields (e.g., De Cesarei et al., 2009; Nummenmaa et al., 2009; Coy and Hutton, 2012). Therefore, we expected that the processing of emotional content might begin before the eyes landed on the target image. This was expected to confound the analyses of brain responses related to eye movements on the target regions. Contrary to these expectations, our results showed similar LPP responses in both presentation conditions. These findings suggest that co-registration of eye movements and EEG is a valid technique to measure brain responses to emotional visual stimuli during free viewing. However, the use of co-registration technique is faced with several technical and data-analytical problems, which are discussed in more detail the following chapters.

#### Ocular artifact correction

Eye movements create large artifacts to EEG recordings (Plöchl et al., 2012). Therefore, co-registration of eye movements and EEG depends on efficient tools for ocular artifact correction. In the present study, we applied a principal component analysis (PCA)-based spatial filter (Ille et al., 2002) to correct for corneoretinal eye movement artifacts. In order to spare brain activity related to the stimulus processing, representative PCA components for eye blink and eye movement artifacts were manually defined outside the experimental trials. The artifact correction was run for continuous data, which then allowed flexible segmentation of the corrected EEG to time-locking points around the first target entries. Moreover, to control for the possibility that the emotional differences in the LPP responses recorded in the parallel condition were due to earlier differences caused by eye movement artifacts, the ERP-amplitudes were analyzed between −50 and 50 ms around the target entry time. These analyses showed no systematic differences between the emotional conditions around the time-locking point, suggesting that the differences in LPPs were not due to early response deviations that could possibly result from oculomotor artifacts.

#### Hardware synchronization

Accurate information about the eye position at a given time is a basic requirement for time-locking the ERP responses with respect to the eye movement events. Because saccades produce large potentials in the electrodes attached close to the eyes (i.e., the electro-oculogram, EOG), these electrodes are suitable for determining the latency of large saccades in the EEG data (Dimigen et al., 2011). However, EOG-data do not provide accurate information about the spatial location of the fixations over the stimulus, while co-registration of EEG and video-based eye-tracking data can measure accurate gaze position with reported spatial resolutions up to 0.01° (Holmqvist et al., 2011). We solved the synchronization between EEG and eye movement data with shared pulses that were sent by the stimulus presentation software to both data sets every few seconds. Other possible problems related to simultaneous recording of video-oculography and EEG, include, for example, the physical contact between EEG sensors and the eye-tracking device. In the present study, a remote eye tracker was used, which allowed a contact-free recording of eye movements. In order to avoid muscle artifacts resulting from head stabilization, participants were comfortably seated in an armchair and their sitting position was stabilized with cushions. The use of active electrodes prevented the electromagnetic fields produced by the eye tracker from disturbing the EEG data. Co-registration of eye movements and EEG is technically challenging, but as concluded also by other authors, the technical problems of hardware synchronization and ocular artifact correction appear to be solvable (see Dimigen et al., 2011; Kliegl et al., 2012).

#### Overlapping potentials

Temporally overlapping potentials evoked by target fixations and the background EEG activity, as well as the temporal overlap between the potentials elicited by successive fixations create another challenge for the co-registration technique. Differences in background EEG activity between EFRPs can be avoided, for instance, by excluding fixations in which background activity is likely to differ (e.g., the first fixation of a trial) (see Dimigen et al., 2011). Selection of fixation subsamples has also been proposed as a solution for overlapping potentials between successive fixations (see Dimigen et al., 2011)^2^. In the present study, the serial condition allowed a full control of the stimuli that were presented at a given time, but required unnaturally long inter-stimulus intervals (2–4 s) to prevent the overlap of subsequent potentials. The effects of overlapping potentials were partly solved by comparing the ERP responses time-locked to first target entries to the results established in the serial condition. These results indicated that processing of emotional content elicited comparable responses between the serial and parallel presentation conditions. The corresponding response latencies and topographies, thus, suggested that the responses in the parallel condition reflected emotional processing and were not resulted by overlapping brain potentials from subsequent fixations or the oculomotor activity caused by the free viewing task. Moreover, the ERP-analysis in the parallel condition were restricted to the first target entries. In these situations, the eyes arrive from neutral images, which at least partly ensured that no ongoing emotional processing of previous images contaminated the responses. More generally, the co-registration technique allows investigations of reinspection events to previously viewed parts of the stimulus or investigations of events where processing is distributed over several fixations (e.g., lag/spill-over effects) (Kliegl et al., 2012). However, such events were not analyzed in the present study.

#### Associated effects between eye movement measures and ERP responses

Previous research suggests that amplitudes of saccades that precede or follow fixations affect the EEG around fixations (Keren et al., 2010). Moreover, differences in fixation durations translate into changes in the phases of overlapping ERP responses (Dimigen et al., 2011). Therefore, several control analyses were performed for the saccade kinematics observed during the parallel condition. First, to control for the possible effects of saccades on ERPs time-locked to the first target entry, we calculated the directions, amplitudes, and durations of incoming saccades for the target images across the emotional conditions. None of these variables accounted for the observed differences in ERPs between emotion conditions.

Moreover, we were interested in a response (LPP) with a timeline that exceeded the fixation duration in most cases. Therefore, it was possible that the ERPs time-locked to the first target entry were contaminated by eye movement artifacts. In order to control for such effects, we calculated the number of within-image saccades and their amplitudes and directions in the 500 ms time window. These analyses revealed more widespread saccades in the neutral than in the unpleasant and pleasant target images. The number of within-image saccades and their directions did not differ between the emotional conditions. These analyses further confirmed that the observed ERP differences in the parallel condition were not due to eye movement artifacts. We hypothesized that longer within-image saccade amplitudes would result in elevated responses for the neutral condition. However, this was not the case. Further, the fact that the ERP responses differed between pleasant and unpleasant condition, while there were no differences in within-image saccade amplitudes between these conditions, supported the conclusion that differences were not due to remains of ocular artifacts.

The single-trial analysis that combined eye movement and ERP measures further suggested that systematic differences in eye movement measures did not explain the observed ERP effects. This analysis revealed no effect of the pre-target saccade amplitudes and the first target fixation duration on ERP-responses at the two studied time windows (125–175 and 400–500 ms).

#### Evaluation of the results and conclusions

Comparison of the ERP results between the serial and parallel presentation conditions suggested elevated responses in the serial condition. The experimental design prevented us from concluding whether the difference was due to the fact that four pictures competed for attentional resources simultaneously in the parallel condition, while in the serial condition, only one picture was attended at a time. Further, the parallel condition allowed parafoveal preview of the pictures, which may have attenuated the responses when the pictures were fixated for the first time. In future studies, these effects can be dissociated by systematically varying the amount of simultaneously presented pictures in the parallel condition.

In the present study, the participants were instructed to look through all four images freely and to respond by clicking a mouse when they were ready to continue onto the next trial. Thus, no explicit instruction to respond as fast as possible was given. This may have influenced the results, because the target entry times (over 1 s) after the trial onset were significantly longer than for example the saccade latencies reported by Nummenmaa et al. (2009). Another possible reason for the relatively long target entry times is that our experimental setup contained more competing distractor images than the previous studies (e.g., Nummenmaa et al., 2006, 2009; Coy and Hutton, 2012).

Further, it is interesting that pleasant and unpleasant conditions differed in the number of fixations before entering the target image, while the target entry times suggested no negativity effects. The participants possibly made longer fixations prior to entering an unpleasant (vs. pleasant) target image, which would explain the lower number of fixations before the target entry, but no difference in the target entry times. The two measures of attentional engagement also showed discrepant results in terms of the negativity effect. That is, the number of fixations suggested that unpleasant images were fixated more frequently than pleasant images, while the dwell times showed no differences between the unpleasant and pleasant conditions. This is a curious finding since the results of attentional orienting toward unpleasant images suggested that longer fixations were made prior to target entry. On the contrary, the results about engagement of attention suggest that after entering the target image the participants made many but shorter fixations on the unpleasant images, whereas the total dwell times were the longest for pleasant images.

Previous studies suggest that differences in tasks and stimulus materials may confound the results (e.g., Lipp et al., 2004). For example, the long-latency LPP response is strongly influenced by arousal (Olofsson et al., 2008). The observed differences in arousal ratings between unpleasant and pleasant conditions in the present study may partly explain the negativity effect in the ERP-results. Furthermore, the heterogeneity in the nature of the emotions depicted in the images may have confounded the results. The present study also adopted a typical paradigm for investigating attention to emotional stimuli. That is, the stimulus displays contained a number of independent images with unrelated contents and locations. Therefore, an independent emotional gist could have been extracted from each image, and the few possible locations where the items could be displayed may have eased the task by increasing the expectation of the emotional stimuli. Thus, it remains unclear whether these effects would remain under more natural conditions where perceptual and foveal load are high, and most importantly where the emotional objects are part of a whole scene. This necessitates that in the future, emotional information processing should be studied with emotional items embedded in the scene (see Acunzo and Henderson, 2011).

In summary, rapid processing of emotional stimuli is a critical aspect of emotional responsiveness. The eye movement results of the present study suggested that emotional content was detected in the parafoveal/peripheral visual field, and was therefore attended faster than neutral information. However, corresponding LPP responses to emotional stimuli were recorded across the SVP and free viewing of emotional scenes. The ERP results, thus, did not show any parafoveal processing effects. Our results were consistent with the view that emotional processing depends on overt attentional resources. Further, the present results suggest that recording of eye movements and ERPs simultaneously provides complementary information about cognitive processing and allows for direct comparisons between neural activity and oculomotor behavior. According to Olofsson et al. (2008) the advantage of collecting behavioral measures simultaneously with ERPs allows us to validate the theoretical interpretations of the ERP results. That is, the behavioral and eye movement data can provide an index of attention revealing the functional significance of waveform modulations by emotional content. Mapping the correlates between behavioral performance measures and affective ERP changes helps to identify the psychological mechanisms underlying affective changes in neuroelectrical responses.

### Conflict of interest statement

The authors declare that the research was conducted in the absence of any commercial or financial relationships that could be construed as a potential conflict of interest.

## Footnotes

^1^Other proposed ocular artifact correction techniques for co-registration studies include, for example, independent component analysis (ICA) (Baccino, 2011), or the surrogate MSEC model (Ille et al., 2002; Dimigen et al., 2011; Kliegl et al., 2012). Furthermore, it should be noted that even though some ocular artifacts remain on the frontal channels, it does not necessarily preclude inspection of the central or posterior channels, since the artifact potentials attenuate with increasing distance to the eyes (see Kretzschmar et al., 2009; Picton et al., 2000).

^2^Mathematical techniques that decompose the effects overlapping potentials also seem promising. For example, the ADJAR-technique (Woldorff, 1993) has been proposed as a method to dissociate the effects of temporally overlapping potentials (Baccino, 2011; Dimigen et al., 2011).

## Appendix

Full list of IAPS images used in the study:

pleasant: 1440, 1460, 1710, 1721, 2057, 4220, 4599, 4659, 4660, 4680, 5010, 5201, 5450, 5460, 5470, 5621, 5623, 5629, 5700, 5831, 5870, 5910, 8021, 8030, 8031, 8034, 8080, 8090, 8161, 8170, 8180, 8190, 8200, 8210, 8300, 8370, 8380, 8400, 8470, 8490, unpleasant: 1300, 3000, 3010, 3030, 3060, 3063, 3064, 3071, 3080, 3100, 3110, 3120, 3130, 3140, 3150, 3170, 3400, 3500, 3530, 6230, 6260, 6300, 6312, 6313, 6350, 6370, 6510, 6540, 6560, 9040, 9050, 9250, 9252, 9405, 9410, 9570, 9600, 9810, 9910, 9921, neutral: 1450, 1670, 2010, 2020, 2190, 2200, 2270, 2500, 2630, 2840, 2870, 2880, 2890, 4653, 4658 5020, 5250, 5390, 5410, 5500, 5510, 5520, 5530, 5531, 5532, 5533, 5534, 5622, 5720, 5731, 5740, 5800, 5900, 6150, 7000, 7002, 7004, 7006, 7009, 7010, 7020, 7030, 7034, 7035, 7040, 7050, 7060, 7080, 7090, 7100, 7140, 7160, 7170, 7175, 7182, 7183, 7185, 7190, 7205, 7207, 7233, 7237, 7238, 7286, 7351, 7352, 7490, 7491, 7500, 7503, 7510, 7550, 7620, 7710, 7820, 7830, 7950, 8311, 8461, 8465
